# Dietary Habits and Depression in Community-Dwelling Chinese Older Adults: Cross-Sectional Analysis of the Moderating Role of Physical Exercise

**DOI:** 10.3390/nu16050740

**Published:** 2024-03-05

**Authors:** Kai Wei, Shaohui Lin, Junjie Yang, Chunbo Li

**Affiliations:** 1 Department of Traditional Chinese Medicine, Shanghai Mental Health Center, Shanghai Jiao Tong University School of Medicine, Shanghai 200030, China; wei.kai@hotmail.com; 2 Shanghai Institute of Traditional Chinese Medicine for Mental Health, Shanghai 201108, China; 3 Shanghai Key Laboratory of Psychotic Disorders, Shanghai Mental Health Center, Shanghai Jiao Tong University School of Medicine, Shanghai 200030, China; shenhui1030@163.com (S.L.); junjieyang@sjtu.edu.cn (J.Y.); 4 Institute of Psychology and Behavioral Science, Shanghai Jiao Tong University, Shanghai 200030, China

**Keywords:** dietary habits, physical exercise, depression, older adults

## Abstract

Background: Healthy diets and physical exercise, two modifiable lifestyle factors, are protective against depression in older adults. This study aimed to investigate whether physical exercise may influence the associations of dietary habits with depression in Chinese community-dwelling older adults. Methods: In the 2018 wave of the Chinese Longitudinal Healthy Longevity Survey, 12,708 community-dwelling older adults aged ≥65 years were included for analyses. Older adults’ dietary habits (including daily intake of food components such as fruits, vegetables, animal oil, and so on) and physical exercise were assessed. Depression was evaluated via the 10 item Center for Epidemiologic Studies Depression (CES-D-10) scale. The influences of physical exercise on the associations of dietary habits with depression were estimated using logistic regression models adjusted for confounders. Results: Older adults who took physical exercise had a significantly decreased probability of depression (adjusted OR = 0.73, *p* < 0.001). As for dietary habits, the intake of fruits, vegetables, eggs, nut products, mushrooms or algae, and vitamins were inversely associated with the prevalence of depression (adjusted ORs = 0.61–0.81; *p*-values: from <0.001 to 0.025), while animal oil was positively associated with it (adjusted OR = 1.52, *p* < 0.001). When stratified by physical exercise, older adults who ate fruits or vegetables had consistent decreased risk of depression, no matter whether they took physical exercise or not (adjusted ORs = 0.52–0.70), while the intake of eggs, nut products, and vitamins were inversely associated, and animal oil was consistently positively associated with depression only in older adults who did not take physical exercise (adjusted ORs = 0.79, 0.68, 0.63, and 1.67, respectively). Conclusions: Physical exercise may conceal the potential protective effects of some healthy dietary habits in terms of depression and counteract the detrimental effects of the unhealthy habits. Some dietary habits may be considered as alternative protective measures for depression in community-dwelling older adults when physical exercise cannot be performed.

## 1. Introduction

Depression is a worldwide public health problem, with a lifetime prevalence of 6.8% in China [1], which is consistently increasing especially after the period of COVID-19 [2], potentially becoming the leading contributor to global disease burden [3]. Population studies have demonstrated that older adults aged ≥ 65 years are more likely to be diagnosed with depression, the prevalence of which being estimated to be 15–20% [4]. When people enter old age, they may face changes in family and social roles, decline in physical and cognitive functions, economic stress, and loss of spouses. If older adults cannot cope with these changes rationally, it may cause negative emotions and a sense of worthlessness, eventually leading to depression [5]. With worldwide population aging gradually becoming an irreversible trend, especially in China, the situation of depression will be increasingly more severe in older adults. A study that involved in 29 cities in China found that more than 39% of older adults had depressive symptoms [6]. Geriatric depression has detrimental impacts on older adults’ quality of life and may further raise their care demands and promote suicidal behaviors, thus bringing about a significant burden to families and society [7].

However, on one hand, depressed older adults are less likely to discuss their mental status or seek professional treatment than young people [4]. On the other hand, even when they try to seek medical treatment, the available monoaminergic antidepressants can only result in the remission of depressive symptoms in about a third of them, and half are still not in remission even after the combined application of different antidepressants [8]. In addition, the accompanied high side effects of antidepressants can cause non-adherence, which was found to influence about 30% of depressed patients within 1 month and up to 60% within 3 months [9]. This is a major challenge and may worsen depressive symptoms or lead to other age-related diseases in older adults [10]. Therefore, optimizing clinically meaningful, non-pharmacological adjunctive therapies for depressed older adults will be important, considering the rapid population ageing and the consequent increasing healthcare demands [4].

Among these non-pharmacological treatments, physical exercise is one of the most commonly used, either alone or in combination with other therapies. Studies have demonstrated the protective role of physical exercise against depression in older adults [11,12,13], the mechanism of which may be improving the brain structural morphology by reducing inflammation, improving the neural network connectivity in multiple brain regions, enhancing neuroplasticity, and so on [11]. The WHO recommends it as a standard supplementary treatment for depression [4]. Despite the strong protective role of physical exercise on depression, studies still recommend considering the synergistic effects of physical exercise in conjunction with other lifestyle factors such as diet, sleep patterns, social interaction, and so on [14]. Healthy dietary habits, a modifiable lifestyle factor, has also been demonstrated to be associated with a decreased risk of depression and less severe depressive symptoms in older adults according to many published studies in the literature [15,16,17], therefore having the potential to be another non-pharmacological adjunctive intervention for depression. With respect to the combination of physical exercise and healthy diet on depression, inconsistent results have been found. One study found no beneficial effect of adding physical exercise to an energy-restricted diet for depression in overweight and obese adults [18], while another study found the joint effect of high dietary quality and physical activity against depression in U.S. adults aged 20 to 80 years [19]. Up until now, it remains unclear as to whether physical exercise and healthy dietary habits may synergistically decrease the risk of depression in Chinese community-dwelling older adults, as well as whether physical exercise may influence the associations of dietary habits with depression.

In the present study, we utilized cross-sectional data from the population-based Chinese Longitudinal Healthy Longevity Survey (CLHLS) conducted in 2018, which included 12,708 older adults aged 65 years or more who were residing in the community for analyses. We examined the sociodemographic, socioeconomic, dietary, physical, and cognitive factors related to physical exercise and depression; assessed the associations of physical exercise and dietary habits with depression; and determined whether physical exercise and dietary habits had synergistic effects on depression in Chinese community-dwelling older adults, as well as whether the associations of dietary habits with depression were different in older adults who engaged in physical exercise or not.

## 2. Materials and Methods

### 2.1. Study Design and Participants

The CLHLS is an ongoing, prospective cohort study conducted in community-dwelling Chinese older adults. It covers most of the provinces in China and aims to investigate factors associated with the healthy longevity of the Chinese population. The study was approved by the Research Ethics Committee of Peking University (IRB00001052-13074), and written informed consent was obtained from the older adults or their proxy respondents. Trained interviewers carry out the survey door-to-door with a structured questionnaire, following the related regulations and guidelines.

In the 2018 wave, 15,874 older adults were initially interviewed. We excluded 2646 participants with no assessment of the 10 item Center for Epidemiologic Studies Depression (CES-D-10) scale or that had 2 items or more missing, 89 participants aged < 65 years, and 431 institutionalized participants, meaning that we ultimately included 12,708 community-dwelling older adults for the analyses.

### 2.2. Measurements

The sociodemographic, socioeconomic, dietary, physical, and cognitive factors related to physical exercise and depression were examined, and the associations of dietary habits with depression in the whole population and those stratified based on physical exercise were assessed. 

#### 2.2.1. Physical Exercise

In our study, physical exercise was assessed via the question “Do you take physical exercises regularly at present?” (referring to purposive fitness activities, e.g., walking, playing ball, running, Qigong), with the answers “yes” or “no”.

#### 2.2.2. Depression

Depressive symptoms of older adults were measured using the 10 item CES-D scale in our study. According to the original research of CES-D-10 [20], only one missing response was allowed in this scale (9 of 10 items complete), which should further be substituted by the mean score of other 9 items. The 10 items were answered in a four-scale metric, from “rarely” (0 point) to “some days” (1 point), “occasionally” (2 points), and “most of the time” (3 points). For the 2 positive questions—“I felt hopeful about the future” and “I was happy”—answers were reversely coded before summation. The total score of the CES-D-10 scale was 30 points, with a higher score indicating greater depressive severity. Depression was defined if a participant scored ≥ 10 points in the CES-D-10. This threshold has been widely utilized in previous studies and has been well-validated for measuring depression in Chinese older adults [21,22]. The internal consistency Cronbach’s α value of this scale was 0.813 in our study, which indicates a reasonable internal consistency level.

#### 2.2.3. Dietary Habits

Dietary habits of older adults were assessed via the question “How often do you eat fruits, vegetables, animal oil, meat, fish, eggs, food made from beans, salt-preserved vegetables, sugar, garlic, milk products, nut products, mushroom or algae, vitamins, and medicinal plants at present?” [23]. The intake of fruits and vegetables included “every day/almost every day”, “quite often”, “occasionally”, and “rarely or never”. We recoded the former two responses as “eat”, and the latter two as “don’t eat”, for the purpose of statistical analysis. The intake levels of other food components were as follows: “almost every day”; “not every day, but at least once per week”; “not every week, but at least once per month”; “not every month, but occasionally”; and “rarely or never”. We also recoded the former two responses as “eat”, and the other responses as “don’t eat”.

#### 2.2.4. Covariates

Sociodemographic characteristics including age, gender, race, education, residence, marital status, BMI, occupation, alcohol drinking, smoking and socioeconomic status were recorded; living preference and arrangement were assessed; and the social/leisure activity score was calculated [23,24]. Self-reported health and interviewer-rated health were assessed by the individuals and by the interviewers. Comorbidity was assessed by determining whether the participant was afflicted with any of the 24 common chronic diseases listed in the questionnaire, such as diabetes, hypertension, heart disease, and stroke, among others. Serious illness in the past 2 years was defined as “illness that causes hospitalization or being bedridden all the year around”. Visual and hearing abilities were also examined. Functional limitation was defined as experiencing difficulty in performing one or more of the Katz Basic Activities of Daily Living (ADL) or Lawton Instrumental Activities of Daily Living (IADL) tasks. The education-adjusted criteria were adopted to define “cognitive impairment” as described in the previous study, using the Chinese version of the Mini-Mental State Examination (MMSE) [23].

### 2.3. Statistical Analysis

Categorical variables in our study were presented as n (%) and quantitative data as means (SD). The χ^2^ test was used to investigate the differences of the categorical variables between groups, and the *t*-test or non-parametric test was used for the quantitative data. To evaluate the relationships between physical exercise and dietary habits with depression in the total sample and the associations of dietary habits with depression stratified by physical exercise, logistic regression models were employed. The corresponding odds ratios (ORs) and 95% confidence intervals (CIs) were also calculated, adjusting for covariates of age, gender, race, education, residence, marital status, BMI, occupation, alcohol drinking, smoking, socioeconomic status, living arrangements, living preference, ≥2 comorbidities, serious illness in the past 2 years, social/leisure activity score, poor interviewer-rated health, hearing problems, visual impairment, functional limitations, and cognitive impairment. The interaction terms between dietary habits and physical exercise for depression were assessed in the logistic regression models adjusted for the same set of confounders cited above. The presence of multicollinearity among the covariates in the aforementioned regression models was evaluated by computing the values of variance inflation factor (VIF, <10 indicating the absence of multicollinearity). The acceptable level of significance was set as two-sided *p* < 0.05. Stata version 14.0 (StataCorp LP, College Station, TX, USA) was used for data analysis.

## 3. Results

### 3.1. Baseline Characteristics by Physical Exercise and Depression

In our study, 33.7% of community-dwelling older adults took physical exercise, and 14.6% had depression (Table 1). Compared with those who did not take physical exercise, older adults who took physical exercise were relatively younger and more likely to be male, married, and living in a city/town, and were less likely to eat animal oil or have hearing/visual impairment, poor self-/interviewer-rated health, functional limitation, cognitive impairment, and depression; more of them were Han Chinese and were currently smoking and drinking alcohol, had ≥1 year education and professional occupations, preferred living alone, and had better socioeconomic status. In addition, more of them ate fruits, vegetables, meat, fish, eggs, food made from beans, garlic, milk and nut products, mushroom or algae, vitamins, and medicinal plants; they also had higher BMIs and social/leisure activity scores. Depressed older adults’ baseline characteristics, however, were mostly on the contrary. Furthermore, there was no difference in race and residence between non-depressed and depressed older adults, and depressed older adults took less physical exercise, were more likely to live alone, and were more likely to have had a serious illness in the past 2 years compared with their non-depressed counterparts. In addition, both older adults who took physical exercise and had depression were more likely to have ≥2 comorbidities.

### 3.2. The Associations between Physical Exercise and Dietary Habits with Depression

As shown in Table 2 and Table 3, physical exercise was found to be inversely associated with the prevalence of depression. Adjusted for confounders including dietary habits, older adults who took physical exercise were still nearly 30% less likely to have depression (OR = 0.73, 95% CI = 0.62–0.86, *p* < 0.001). Some dietary habits were also inversely associated with depression, including fruits (OR = 0.65, 95% CI = 0.56–0.75, *p* < 0.001), vegetables (OR = 0.60, 95% CI = 0.49–0.73, *p* < 0.001), eggs (OR = 0.81, 95% CI = 0.70–0.94, *p* = 0.004), nut products (OR = 0.69, 95% CI = 0.56–0.84, *p* < 0.001), mushroom or algae (OR = 0.78, 95% CI = 0.64–0.94, *p* = 0.010), and vitamins (OR = 0.68, 95% CI = 0.55–0.86, *p* = 0.001), while animal oil was positively associated with depression (OR = 1.53, 95% CI = 1.25–1.88, *p* < 0.001), adjusting for confounders including physical exercise.

### 3.3. The Role of Physical Exercise in the Associations between Dietary Habits and Depression

Some dietary habits and physical exercise showed synergistic effects on depression, indicated by the lower OR of depression in those who took physical exercise as well as ate fruits, vegetables, eggs, food made from beans, salt-preserved vegetables, milk and nut products, mushroom or algae, vitamins, and medical plants, compared with other combinations of physical exercise and dietary habits (Table 3). When stratified by physical exercise, older adults who ate fruits or vegetables had consistently decreased risk of depression, no matter whether they took physical exercise or not (ORs = 0.52–0.70, *p* values: from <0.001 to 0.016), while eggs, nut products, and vitamins were inversely associated (OR = 0.79, 95% CI = 0.67–0.94, *p* = 0.007; OR = 0.68, 95% CI = 0.53–0.88, *p* = 0.003; OR = 0.63, 95% CI = 0.47–0.85, *p* = 0.002, respectively) and animal oil was positively associated (OR = 1.67, 95% CI = 1.34–2.09, *p* < 0.001) with depression only in older adults who did not take physical exercise (Table 3). The interaction effects between physical exercise and dietary habits for depression were not found to be significant.

## 4. Discussion

In our study, older adults who took physical exercise were younger; more likely to be male, married, and live in city/town with better education and socioeconomic status; had more social/leisure activities and better physical/cognitive functions; and were less likely to be depressed, compared with those who did not. Older adults who had depression, however, were mostly on the contrary. These findings indicated that physical exercise was more prevalent and would be more feasible in the young old males who were healthier, wealthier, and more outgoing. In the logistic regression models, physical exercise was found to be inversely associated with depression in community-dwelling older adults, indicated by OR without adjustment (=0.47), as well as adjusting for sociodemographic, socioeconomic, physical, and cognitive health statuses (adjusted OR = 0.68), as well as dietary habits (adjusted OR = 0.73).

Depression is a complicated disease characterized by a wide range of psychological and physiological symptoms, including persistent low mood, anhedonia, emotional dysregulation, and cognitive impairment [25]. Elevated inflammation, oxidative stress, and mitochondrial dysfunction may contribute to impaired neuroplasticity and reduced neurogenesis, both of which are core elements of the neurobiology of depression [26]. Physical exercise has the potential to have antidepressant effects and has been recommended by the WHO as a standard supplementary treatment for depression [4]. It improves the brain structural morphology by restoring the expression of neurotrophic factors, reducing inflammation, positively affecting oxidative stress, improving the neural network connectivity in multiple brain regions, enhancing neuroplasticity, and improving brain neuroprocessing in depressed patients, thereby promoting positive emotions and reducing depression [11]. 

Two meta-analyses compared the antidepressant effects of three broad exercise modes, namely, mind–body, aerobic exercise, and resistance exercise, finding that the mind–body exercise had the largest improvement on depressive symptoms in older adults, followed by aerobic exercise [4,11]. There are significant physiological, mechanical, and metabolic distinctions among these exercise modes. Mind–body exercise encompasses a variety of low-impact and intentionally slow movements, along with breathing, meditation, and progressive relaxation, which are more suitable for older adults, especially those with various forms of functional decline [4]. This exercise combines low-intensity muscular activities, such as balance training or flexibility, with an internally focused approach that promotes a contemplative mental state [27]. Simultaneously engaging in physical and mental exercises empowers older adults to manage their depressive symptoms by regulating their internal emotional states, potentially offering added advantages compared with other exercise modes [11]. Aerobic exercise represents a unique type of activity that engages large muscle groups, such as in the rhythmic movement of body mass during activities of varying intensities (e.g., walking, jogging, or running). This activity is accompanied by an increased metabolic demand, requiring corresponding efforts in breathing, heart rate, and blood flow to match the intensity of exertion [4]. Aerobic exercise can change the expression of monoamine neurotransmitters, increase the level of 5-hydroxytryptamine (5-HT) and norepinephrine, and decrease the secretion of cortisol, thereby alleviating depressive symptoms [28]. By contrast, although resistance exercise may exert antidepressant effects via regulating the expression of monoamine transmitters and neuroimmune indicators [29], it can be challenging to implement in real-life settings for older adults, as once the body has adapted to one intensity, the intensity will need to continually be adjusted to achieve an effective impact [30]. It is worth noting that among these three exercise modes, aerobic exercise had the lowest while mind–body exercise showed the highest dropout rates in randomized controlled trials of depressed older adults, indicating that aerobic exercise may have the highest adherence in the treatment of depression [4]. In our study, physical exercises taken by older adults were referred to as “purposive fitness activities, such as walking, playing ball, running, and Qigong”, among which the former three examples could be categorized as aerobic exercise. Qigong, a type of mind–body exercise, emphasizes slow and gentle movements, deep breathing, and sensory-motor training, which demand a high level of physical fundamentals including strength [11]. Although without accurate parameters of physical exercise such as classification, frequency, intensity, and duration in our analyses, these purposive fitness activities were still significantly associated with a lower risk of depression in community-dwelling older adults. 

Despite the strong protective role of physical exercise on depression, studies still recommend considering the synergistic effects of physical exercise in conjunction with other lifestyle factors such as diet, sleep patterns, social interaction, and so on [14]. Thus, we further investigated the role of dietary habits on depression in community-dwelling older adults, finding that daily intake of fruits, vegetables, eggs, nut products, mushrooms or algae, and vitamins were also inversely associated with depression, whereas intake of animal oil was positively associated with it, adjusting for confounding variables including physical exercise, partially consistent with a previous study [17]. When considering the combinations of physical exercise and dietary habits, physical exercise and daily intake of fruits, vegetables, eggs, foods made from beans, salt-preserved vegetables, milk products, nut products, mushrooms or algae, vitamins, and medical plants were synergistically associated with decreased risk of depression, indicated by the smallest ORs compared with other combinations, although some of the above dietary habits were not significantly associated with depression when analyzed individually. These findings indicated that, in addition to physical exercise, some healthy dietary habits also had the potential to be a non-pharmacological adjunctive intervention for depression in older adults. When considering the protective role of lifestyle factors on depression, both physical exercise and healthy dietary habits should be adopted, so as to achieve multiplying effects.

A healthy diet is a modifiable factor that can positively impact mental health, mood, and cognitive performance, as demonstrated by previous studies. This is because the composition, structure, and function of the brain depend on the availability of essential nutrients [3,31,32]. Research has shown that following a diverse diet rich in fruits, vegetables, nuts, fish, and olive oil—characteristics of the Mediterranean diet—may provide protection against depression [32]. Nutrients such as dietary carotenoids (e.g., lutein and zeaxanthin), commonly found in fruits and vegetables, may accumulate in the human brain and promote brain health, thereby influencing mood and mental well-being [33]. In terms of the mechanisms underlying the potential impact of healthy diets on depression, regulating gut microbiota and reducing oxidative stress and inflammation in the brain may serve as crucial mediating pathways [34,35]. Microorganisms present in the gut communicate with the brain via the gut–brain axis, thereby influencing emotional behaviors and neurological processes [36]. Certain gut bacteria can metabolize carotenoids into various beneficial compounds with enhanced antioxidant properties [37], and these carotenoids may, in turn, impact the composition and diversity of the gut microbiota [38]. Tryptophan found in eggs can be converted to serotonin to regulate mood, and egg yolk, being a good source of vitamin D, plays a crucial role in alleviating symptoms in older depressed patients [39,40]. Additionally, vitamins C and E possess direct free radical scavenging properties that can help alleviate oxidative stress in the brain [41]. Nuts are nutrient-dense foods containing vitamins, monounsaturated fatty acids, and polyunsaturated fatty acids, which are associated with reduced oxidative stress and inflammation, as well as a decreased risk of depression [42]. Fiber and nutrients derived from nuts can also optimize the gut microbiota [43]. Mushrooms are rich in vitamins (e.g., B1, B2, B12, and C), potent antioxidants such as glutathione and ergothioneine, and anti-inflammatory agents, all of which may mitigate depression through their antioxidant and neuroprotective properties [44]. The increased consumption of seaweed and mushroom fiber was found to be inversely correlated with the depressive severity, and seaweed fiber intake was also inversely associated with clinical diagnoses of depression, owing to its anti-inflammatory and antioxidant activities [45]. Conversely, western-style diets, which are high in saturated fat and added sugar, are associated with reduced neurogenesis, increased free radical production, and increased inflammation in the brain, thereby carrying an elevated risk of depression [46].

In the stratification analyses based on physical exercise, we found that fruits and vegetables were consistently inversely associated with depression in older adults, no matter whether they took physical exercise or not, while eggs, nut products, and vitamins were inversely associated with depression and animal oil remained being positively associated with depression only in older adults who did not take physical exercise. These findings were not surprising, as the adjusted ORs of fruits and vegetables for depression (0.67 and 0.61, respectively) were even lower than that of physical exercise (adjusted OR = 0.68) in our study, while the adjusted ORs of eggs, nut products, and vitamins (0.81, 0.71, and 0.71, respectively) were slightly higher. This indicated that daily intake of fruits and vegetables may be an even stronger protective factor against depression than physical exercise in Chinese community-dwelling older adults. The existence of physical exercise may conceal the potential protective effects of those healthy dietary habits with weaker protective effects on depression, as well as counteract the detrimental effects of the unhealthy dietary habits. 

Our study revealed that physical exercise could partially influence the associations of certain daily diet components with depression among community-dwelling older adults. The study encompassed a large sample size covering most of the provinces in China, ensuring the representativeness of our findings. However, several limitations still existed in our study. First, this was a cross-sectional study, and no causal relationship between physical exercise/dietary habits and depression can be ascertained. Second, the categories of physical exercise in our study were not accurately classified, and the frequency, intensity, and duration of the purposive fitness activities were also not recorded, although we assumed that they were aerobic or mind–body exercises fit for older adults. These parameters would be important, as previous studies have found inconsistent results in the associations between the physical activities of different intensities and depression in older adults [12,13]. Third, in our study, dietary habits were assessed only qualitatively, lacking quantitative assessment, which may have resulted in inadequate evidence to guide the daily diets for older adults with depression. Fourth, although previous studies recommend considering the overall diet rather than individual components [17,47], our study still assessed dietary habits based on individual components. This was due to the fact that, in everyday situations, foods are typically consumed in complex and diverse combinations by older adults in China, where people residing in different regions exhibit significantly varied dietary cultures and lifestyles. In the future, longitudinal cohort studies should be conducted, and physical exercise should be assessed in terms of detailed category, frequency, intensity, and duration, in order to identify the optimal exercise prescriptions that are protective against depression and suitable for older adults. Additionally, dietary habits should also be quantitatively assessed to guide the daily diets of older adults, and healthy dietary patterns should be developed to evaluate dietary health across different regions of China, considering the specific dietary cultures and lifestyles. 

## 5. Conclusions

Physical exercise may conceal the potential protective effects of some healthy dietary habits for depression and counteract the detrimental effects of unhealthy habits. Some dietary habits may be considered as alternative protective measures for depression in community-dwelling older adults when physical exercised is unable to be performed.

## Figures and Tables

**Table 1:** Baseline characteristics by physical exercise and depression.

Characteristics	Total Sample	No PE	PE	*p*	NC	Depression	*p*
	(N = 12,708)	8295 (66.3)	4220 (33.7)		10,854 (85.4)	1854 (14.6)	
Sociodemographic							
Age (years) *	83.5 (11.1)	85.1 (11.3)	80.3 (10.0)	<0.001	83.3 (11.1)	85.0 (11.0)	<0.001
Gender (female)	6829 (53.7)	4765 (57.4)	1947 (46.1)	<0.001	5670 (52.2)	1159 (62.5)	<0.001
Race (minority)	660 (6.0)	500 (7.0)	154 (4.2)	<0.001	548 (5.9)	112 (6.9)	0.097
Marital status (SDW)	6905 (54.9)	4853 (59.1)	1928 (46.1)	<0.001	5734 (53.3)	1171 (64.0)	<0.001
Residence (rural)	5714 (45.0)	4149 (50.0)	1488 (35.3)	<0.001	4855 (44.7)	859 (46.3)	0.200
Occupation (professional)	1231 (11.4)	536 (7.7)	674 (18.4)	<0.001	1133 (12.3)	98 (6.2)	<0.001
Education (≥1 year)	5896 (54.4)	3265 (46.5)	2562 (69.7)	<0.001	5203 (56.2)	693 (43.6)	<0.001
BMI (kg/m^2^) *	22.6 (5.8)	22.3 (6.0)	23.3 (5.4)	<0.001	22.7 (5.5)	22.2 (7.4)	<0.001
Current smoker	2046 (16.3)	1253 (15.2)	761 (18.3)	<0.001	1796 (16.7)	250 (13.6)	0.001
Current alcohol drinker	1939 (15.5)	1184 (14.5)	752 (18.1)	<0.001	1743 (16.3)	196 (10.8)	<0.001
Living alone	2179 (17.4)	1427 (17.5)	709 (17.0)	0.494	1762 (16.5)	417 (22.9)	<0.001
Prefer living alone	6295 (50.9)	3825 (47.4)	2376 (57.7)	<0.001	5458 (51.6)	837 (46.9)	<0.001
Socioeconomic status							
Sufficient financial support	10,908 (86.4)	6977 (84.7)	3777 (90.0)	<0.001	9617 (89.2)	1291 (70.3)	<0.001
Economic independence	4433 (35.9)	2423 (29.9)	1952 (48.1)	<0.001	3966 (37.6)	467 (25.9)	<0.001
Adequate medical service	12,239 (97.4)	7970 (97.1)	4098 (98.1)	0.001	10,514 (98.2)	1725 (93.7)	<0.001
Public medical payment	7028 (57.4)	4465 (56.0)	2446 (59.9)	<0.001	6053 (57.9)	975 (54.6)	0.009
Dietary habits							
Fruits	5854 (46.2)	3449 (41.7)	2308 (54.8)	<0.001	5266 (48.6)	588 (31.8)	<0.001
Vegetables	11,448 (90.3)	7327 (88.5)	3952 (93.8)	<0.001	9916 (91.5)	1532 (82.8)	<0.001
Animal oil	1315 (10.4)	1013 (12.2)	278 (6.6)	<0.001	1023 (9.5)	292 (15.8)	<0.001
Meat	9798 (78.1)	6292 (76.7)	3364 (80.7)	<0.001	8462 (78.9)	1336 (73.0)	<0.001
Fish	6027 (48.0)	3786 (46.2)	2162 (51.9)	<0.001	5252 (49.0)	775 (42.4)	<0.001
Eggs	9134 (72.8)	5809 (70.8)	3211 (77.0)	<0.001	7941 (74.1)	1139 (65.2)	<0.001
Food made from beans	6510 (51.9)	4094 (49.9)	2330 (55.9)	<0.001	5694 (53.2)	816 (44.6)	<0.001
Salt-preserved vegetables	3845 (30.7)	2509 (30.6)	1286 (30.9)	0.773	3298 (30.8)	574 (29.9)	0.446
Sugar	3637 (29.0)	2373 (29.0)	1210 (29.0)	0.938	3136 (29.3)	501 (27.4)	0.099
Garlic	5817 (46.4)	3434 (41.9)	2302 (55.3)	<0.001	5077 (47.4)	740 (40.5)	<0.001
Milk products	4907 (39.2)	2813 (34.4)	2022 (48.6)	<0.001	4281 (40.0)	626 (34.3)	<0.001
Nut products	2453 (19.6)	1206 (14.7)	1209 (29.0)	<0.001	2241 (20.9)	212 (11.6)	<0.001
Mushroom or algae	2446 (19.5)	1191 (14.6)	1221 (29.3)	<0.001	2217 (20.7)	229 (12.5)	<0.001
Vitamins	1592 (12.7)	814 (10.0)	753 (18.1)	<0.001	1416 (13.2)	176 (9.6)	<0.001
Medicinal plants	928 (7.4)	394 (4.8)	524 (12.6)	<0.001	837 (7.8)	91 (5.0)	<0.001
Physical and cognitive health status							
Social/leisure activity score (point) *	4.1 (3.3)	3.5 (3.0)	5.5 (3.4)	<0.001	4.3 (3.3)	3.0 (2.9)	<0.001
Physical exercise	4220 (33.7)	-	-	-	3843 (35.9)	377 (20.8)	<0.001
Poor self-reported health	1673 (13.2)	1259 (15.2)	394 (9.3)	<0.001	978 (9.0)	695 (37.6)	<0.001
Poor interviewer-rated health	1533 (12.2)	1296 (15.7)	218 (5.2)	<0.001	925 (8.6)	608 (33.1)	<0.001
Comorbidities (≥2)	5724 (45.3)	3540 (42.9)	2104 (50.1)	<0.001	4755 (44.0)	969 (52.5)	<0.001
Serious illness in the past 2 years	3045 (25.1)	1945 (24.6)	1051 (26.0)	0.097	2453 (23.7)	592 (33.6)	<0.001
Hearing problem	4302 (34.0)	3092 (37.5)	1138 (27.1)	<0.001	3496 (32.4)	806 (43.8)	<0.001
Visual impairment	1889 (15.0)	1490 (18.1)	364 (8.7)	<0.001	1426 (13.3)	463 (25.2)	<0.001
Functional limitation	5521 (43.5)	4248 (51.2)	1185 (28.2)	<0.001	4690 (40.5)	1131 (61.1)	<0.001
Cognitive impairment	1497 (13.8)	1223 (17.4)	257 (7.0)	<0.001	1135 (12.3)	362 (22.8)	<0.001
Depression	1854 (14.6)	1439 (17.4)	377 (8.9)	<0.001	-	-	-

Note. Data presented as n (%) or mean (SD). PE: physical exercise; NC: normal control; SDW, separated/divorced/widowed; BMI: body mass index. * Kruskal–Wallis test was used, and data are presented as mean (SD); for other characteristics, the χ^2^ test was used, and data are presented as n (%), similarly hereinafter.

**Table 2:** The association between physical exercise and depression in the total sample.

	N with/without Depression	Model 1	Model 2	Model 3
No physical exercise	1439/6856	1.0 (reference)	1.0 (reference)	1.0 (reference)
Physical exercise	377/3843	0.47 (0.41–0.53) *p* < 0.001	0.68 (0.58–0.80) *p* < 0.001	0.73 (0.62–0.86) *p* < 0.001

Model 1: no adjustment. Model 2: adjusted for age, gender, race, marital status, residence, education, occupation, BMI, smoking, alcohol drinking, socioeconomic status, living arrangements, living preference, comorbidities (≥2), serious illness in the past 2 years, social/leisure activity score, poor interviewer-rated health, hearing problem, visual impairment, functional limitation, and cognitive impairment. Model 3: adjusted for covariates in Model 2, as well as dietary habits.

**Table 3:** The associations between dietary habits and depression in the total sample and stratified by physical exercise.

	Don’t Eat		Eat		OR (95% CI) for the Associations between Dietary Habits and Depression within Each Stratum of Physical Exercise
	N with/without Depression	OR (95% CI) *	N with/without Depression	OR (95% CI) *	
Fruits	1263/5561	1.0 (reference)	588/5266	0.67 (0.58–0.78) *p* < 0.001	
No physical exercise	989/3833	1.0 (reference)	448/3001	0.70 (0.59–0.82) *p* < 0.001	0.66 (0.56–0.79) *p* < 0.001
Physical exercise	245/1662	0.73 (0.60–0.90) *p* = 0.003	131/2177	0.45 (0.36–0.57) *p* < 0.001	0.70 (0.52–0.94) *p* = 0.016
Vegetables	319/918	1.0 (reference)	1532/9916	0.61 (0.50–0.74) *p* < 0.001	
No physical exercise	266/687	1.0 (reference)	1171/6156	0.64 (0.51–0.79) *p* < 0.001	0.64 (0.51–0.79) *p* < 0.001
Physical exercise	45/216	0.84 (0.53–1.32) *p* = 0.448	331/3621	0.43 (0.33–0.55) *p* < 0.001	0.52 (0.33–0.81) *p* = 0.004
Animal oil	1559/9801	1.0 (reference)	292/1023	1.52 (1.24–1.86) *p* < 0.001	
No physical exercise	1193/6069	1.0 (reference)	244/769	1.59 (1.27–1.98) *p* < 0.001	1.67 (1.34–2.09) *p* < 0.001
Physical exercise	333/3598	0.71 (0.60–0.84) *p* < 0.001	44/234	0.88 (0.54–1.41) *p* = 0.587	1.02 (0.61–1.72) *p* = 0.929
Meat	494/2257	1.0 (reference)	1336/8462	1.04 (0.88–1.22) *p* = 0.670	
No physical exercise	395/1514	1.0 (reference)	1025/5267	1.05 (0.87–1.26) *p* = 0.614	1.02 (0.85–1.23) *p* = 0.820
Physical exercise	90/715	0.72 (0.52–0.99) *p* = 0.046	284/3080	0.72 (0.57–0.90) *p* = 0.004	1.02 (0.73–1.42) *p* = 0.925
Fish	1055/5466	1.0 (reference)	775/5252	1.08 (0.94–1.24) *p* = 0.276	
No physical exercise	831/3584	1.0 (reference)	590/3196	1.11 (0.94–1.30) *p* = 0.217	1.06 (0.91–1.25) *p* = 0.461
Physical exercise	203/1804	0.72 (0.58–0.90) *p* = 0.003	170/1992	0.73 (0.58–0.92) *p* = 0.008	1.12 (0.85–1.49) *p* = 0.417
Eggs	638/2779	1.0 (reference)	1193/7941	0.81 (0.70–0.94) *p* = 0.005	
No physical exercise	502/1894	1.0 (reference)	919/4890	0.81 (0.68–0.96) *p* = 0.015	0.79 (0.67–0.94) *p* = 0.007
Physical exercise	122/835	0.70 (0.53–0.93) *p* = 0.014	252/2959	0.56 (0.45–0.70) *p* < 0.001	0.91 (0.67–1.23) *p* = 0.532
Food made from beans	1015/5061	1.0 (reference)	816/5694	0.90 (0.79–1.04) *p* = 0.144	
No physical exercise	790/3315	1.0 (reference)	631/3463	0.92 (0.79–1.08) *p* = 0.302	0.90 (0.77–1.06) *p* = 0.209
Physical exercise	205/1630	0.72 (0.58–0.89) *p* = 0.003	169/2161	0.61 (0.49–0.77) *p* < 0.001	0.96 (0.72–1.27) *p* = 0.764
Salt-preserved vegetables	1283/7417	1.0 (reference)	547/3298	0.97 (0.84–1.12) *p* = 0.690	
No physical exercise	994/4698	1.0 (reference)	427/2082	1.02 (0.86–1.20) *p* = 0.857	1.03 (0.87–1.22) *p* = 0.741
Physical exercise	265/2618	0.73 (0.60–0.88) *p* = 0.001	109/1177	0.62 (0.47–0.81) *p* = 0.001	0.83 (0.61–1.12) *p* = 0.215
Sugar	1329/7577	1.0 (reference)	501/3136	1.02 (0.88–1.18) *p* = 0.803	
No physical exercise	1030/4794	1.0 (reference)	390/1983	1.03 (0.86–1.22) *p* = 0.774	1.03 (0.87–1.23) *p* = 0.721
Physical exercise	273/2687	0.70 (0.58–0.84) *p* < 0.001	101/1109	0.70 (0.53–0.92) *p* = 0.010	0.98 (0.72–1.34) *p* = 0.911
Garlic	1088/5638	1.0 (reference)	740/5077	0.95 (0.83–1.10) *p* = 0.509	
No physical exercise	878/3889	1.0 (reference)	540/2894	0.92 (0.78–1.08) *p* = 0.295	0.93 (0.79–1.09) *p* = 0.385
Physical exercise	184/1680	0.65 (0.52–0.81) *p* < 0.001	190/2112	0.69 (0.55–0.86) *p* = 0.001	1.09 (0.82–1.44) *p* = 0.555
Milk products	1200/6423	1.0 (reference)	626/4281	0.96 (0.83–1.12) *p* = 0.598	
No physical exercise	960/4417	1.0 (reference)	456/2357	1.01 (0.85–1.20) *p* = 0.932	0.95 (0.80–1.14) *p* = 0.603
Physical exercise	219/1923	0.75 (0.61–0.92) *p* = 0.006	155/1867	0.63 (0.50–0.80) *p* < 0.001	0.97 (0.72–1.30) *p* = 0.848
Nut products	1614/8462	1.0 (reference)	212/2241	0.71 (0.58–0.87) *p* = 0.001	
No physical exercise	1285/5697	1.0 (reference)	131/1075	0.71 (0.55–0.91) *p* = 0.006	0.68 (0.53–0.88) *p* = 0.003
Physical exercise	299/2658	0.71 (0.59–0.85) *p* < 0.001	75/1134	0.51 (0.37–0.69) *p* < 0.001	0.80 (0.56–1.12) *p* = 0.193
Mushroom or algae	1597/8486	1.0 (reference)	229/2217	0.80 (0.66–0.97) *p* = 0.025	
No physical exercise	1261/5734	1.0 (reference)	155/1036	0.87 (0.69–1.10) *p* = 0.237	0.78 (0.65–1.04) *p* = 0.107
Physical exercise	305/2642	0.74 (0.62–0.88) *p* = 0.001	69/1152	0.51 (0.37–0.69) *p* < 0.001	0.76 (0.53–1.07) *p* = 0.115
Vitamins	1649/9277	1.0 (reference)	176/1416	0.71 (0.57–0.90) *p* = 0.004	
No physical exercise	1299/6066	1.0 (reference)	116/689	0.65 (0.49–0.87) *p* = 0.004	0.63 (0.47–0.85) *p* = 0.002
Physical exercise	315/3097	0.68 (0.58–0.81) *p* < 0.001	59/694	0.57 (0.40–0.81) *p* = 0.002	0.93 (0.64–1.35) *p* = 0.702
Medicinal plants	1735/9858	1.0 (reference)	91/837	0.96 (0.72–1.28) *p* = 0.773	
No physical exercise	1358/6429	1.0 (reference)	58/336	0.99 (0.68–1.45) *p* = 0.975	0.92 (0.63–1.34) *p* = 0.666
Physical exercise	341/3300	0.70 (0.59–0.83) *p* < 0.001	33/491	0.64 (0.42–0.98) *p* = 0.039	1.06 (0.68–1.67) *p* = 0.797

Interaction effects between physical exercise and dietary habits for depression: fruits: OR (95% CI) = 0.89 (0.65–1.22), *p* = 0.456; vegetables: OR (95% CI) = 0.80 (0.50–1.29), *p* = 0.358; animal oil: OR (95% CI) = 0.77 (0.45–1.32), *p* = 0.346; meat: OR (95% CI) = 0.95 (0.66–1.37), *p* = 0.785; fish: OR (95% CI) = 0.91 (0.67–1.24), *p* = 0.562; eggs: OR (95% CI) = 0.98 (0.70–1.37), *p* = 0.918; food made from beans: OR (95% CI) = 0.93 (0.68–1.26), *p* = 0.628; salt-preserved vegetables: OR (95% CI) = 0.84 (0.60–1.17), *p* = 0.297; sugar: OR (95% CI) = 0.98 (0.69–1.37), *p* = 0.888; garlic: OR (95% CI) = 1.16 (0.85–1.58), *p* = 0.342; milk products: OR (95% CI) = 0.84 (0.61–1.14), *p* = 0.278; nut products: OR (95% CI) = 1.01 (0.67–1.51), *p* = 0.977; mushroom or algae: OR (95% CI) = 0.79 (0.53–1.17), *p* = 0.242; vitamins: OR (95% CI) = 1.28 (0.81–2.03), *p* = 0.294; medicinal plants: OR (95% CI) = 0.92 (0.52–1.62), *p* = 0.774. Adjusted for age, gender, race, marital status, residence, education, occupation, BMI, smoking, alcohol drinking, socioeconomic status, living arrangements, living preference, comorbidities (≥2), serious illness in the past 2 years, social/leisure activity score, poor interviewer-rated health, hearing problems, visual impairment, functional limitation, and cognitive impairment. Physical exercise was also adjusted for when investigating the associations of dietary habits with depression in the total sample. * Note: OR (95% CI): odds ratio (95% confidence interval).

## Data Availability

The datasets used and analyzed during the current study are available from the Peking University Open Research Data Platform (https://opendata.pku.edu.cn/dataverse/CHADS?from=timeline&isappinstalled=0, accessed on 7 March 2020).
